# Improvement of Posterior Cruciate Ligament Buckling and Anterior Tibial Subluxation by Anterior Scar Removal of the Posterior Cruciate Ligament: A Case Report

**DOI:** 10.7759/cureus.68074

**Published:** 2024-08-28

**Authors:** Hiroji Fukuta

**Affiliations:** 1 Orthopedics, Fukuta Orthopedics Clinic, Kasugai, JPN

**Keywords:** arthroscopic surgery, intercondylar notchplasty, anterior tibial subluxation, posterior cruciate ligament buckling, graft impingement, anterior cruciate ligament reconstruction

## Abstract

Posterior cruciate ligament (PCL) buckling and anterior tibial subluxation are observed in patients with insufficient anterior cruciate ligament (ACL). Here, we report the case of a patient after ACL reconstruction in whom these symptoms were improved by anterior scar resection of buckled PCL.

The patient was a 46-year-old man. Six years ago, he underwent ACL reconstruction; however, his condition was not satisfactory. Magnetic resonance imaging (MRI) showed intercondylar impingement of the graft, anterior tibial subluxation, and PCL buckling. Intercondylar notchplasty and resection of the anterior scar of PCL were performed arthroscopically. Postoperative MRI showed improvement in PCL buckling and anterior tibial subluxation. His symptoms improved, and he was able to jog one year after surgery.

Anterior scar resection of PCL may improve PCL buckling and anterior tibial subluxation after ACL reconstruction.

## Introduction

Posterior cruciate ligament (PCL) buckling where the PCL curves posterosuperiorly [1-4] and anterior tibial subluxation during passive knee maximum extension (ATS) [5-11] are observed in some anterior cruciate ligament (ACL)-insufficient knees. Herein, we report a case in which PCL buckling and ATS improved when the scar anterior to the PCL was resected to reduce graft impingement six years after ACL reconstruction.

## Case presentation

**Figure 1:**
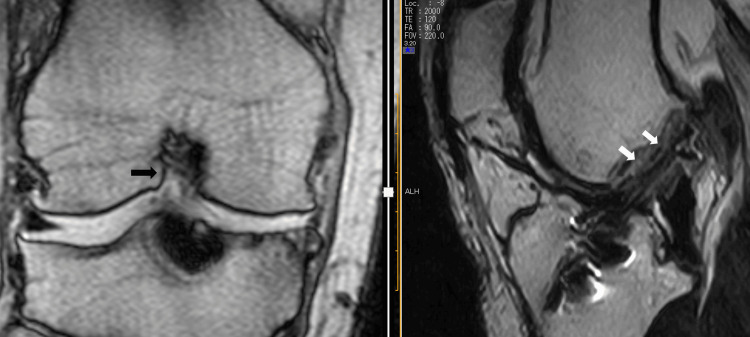
MRI showed narrowing of the lateral intercondylar notch (black arrow) and impingement of the graft (white arrows). MRI: magnetic resonance imaging

The patient was a 46-year-old man. He underwent double-bundle ACL repair six years ago. However, he experienced pain and discomfort after the surgery. His symptoms did not improve, and he was admitted to our clinic with a condition that interfered with his daily life. The range of motion of the right knee joint is 0-140 degrees. The anterior drawer test and Lachman test were negative. The anterior displacement distances measured using the KT-1000 knee arthrometer were 6 and 4 mm on the right and left sides, respectively. There were pain and catching during knee extension loading. They were especially strong with the pivot shift technique. Magnetic resonance imaging (MRI) showed lateral intercondylar notch narrowing and graft impingement (Figure 1).

**Figure 2:**
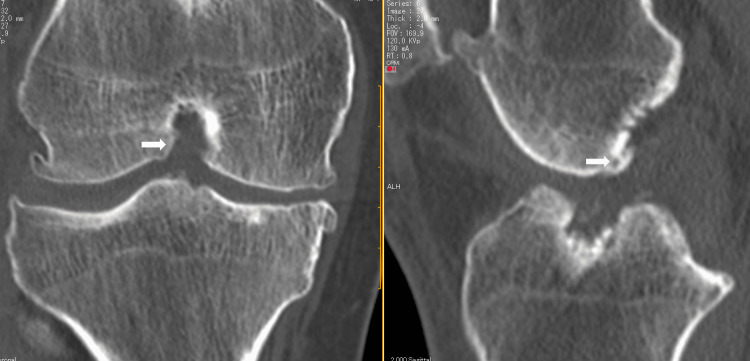
CT showed that the tibial bone hole is blocked by osteophytes outside the intercondylar notch (white arrow). CT: computed tomography

Computed tomography (CT) showed that the bone hole of the graft on the tibial side was partially blocked by the intercondylar wall (Figure 2).

**Figure 3:**
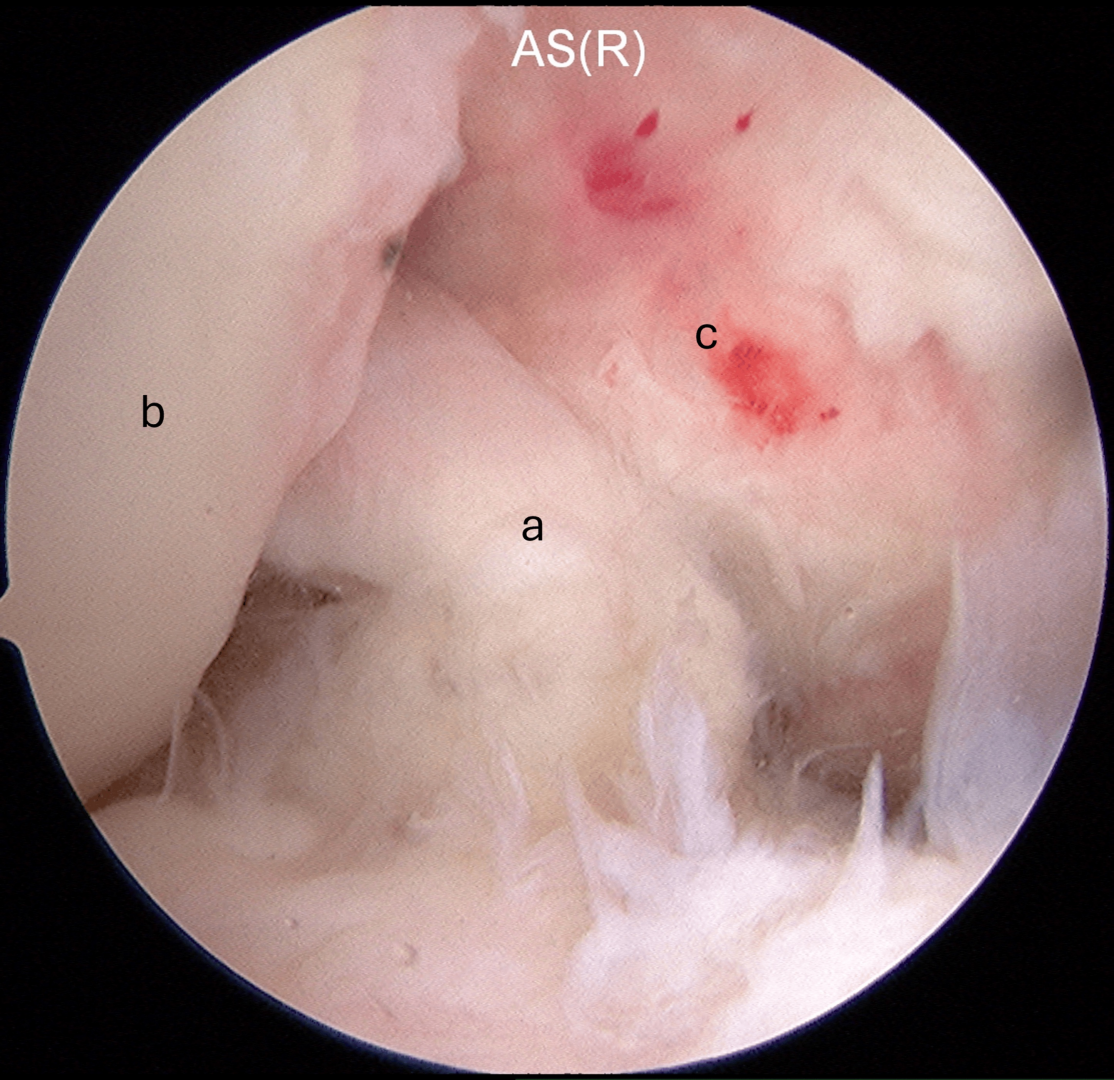
The graft was impinged on the lateral wall of the intercondylar notch and PCL: (a) the graft, (b) the lateral wall of the intercondylar notch, and (c) the PCL. PCL: posterior cruciate ligament

Arthroscopic surgery was performed. The graft was impinging on the lateral intercondylar fossa and PCL (Figure 3).

**Figure 4:**
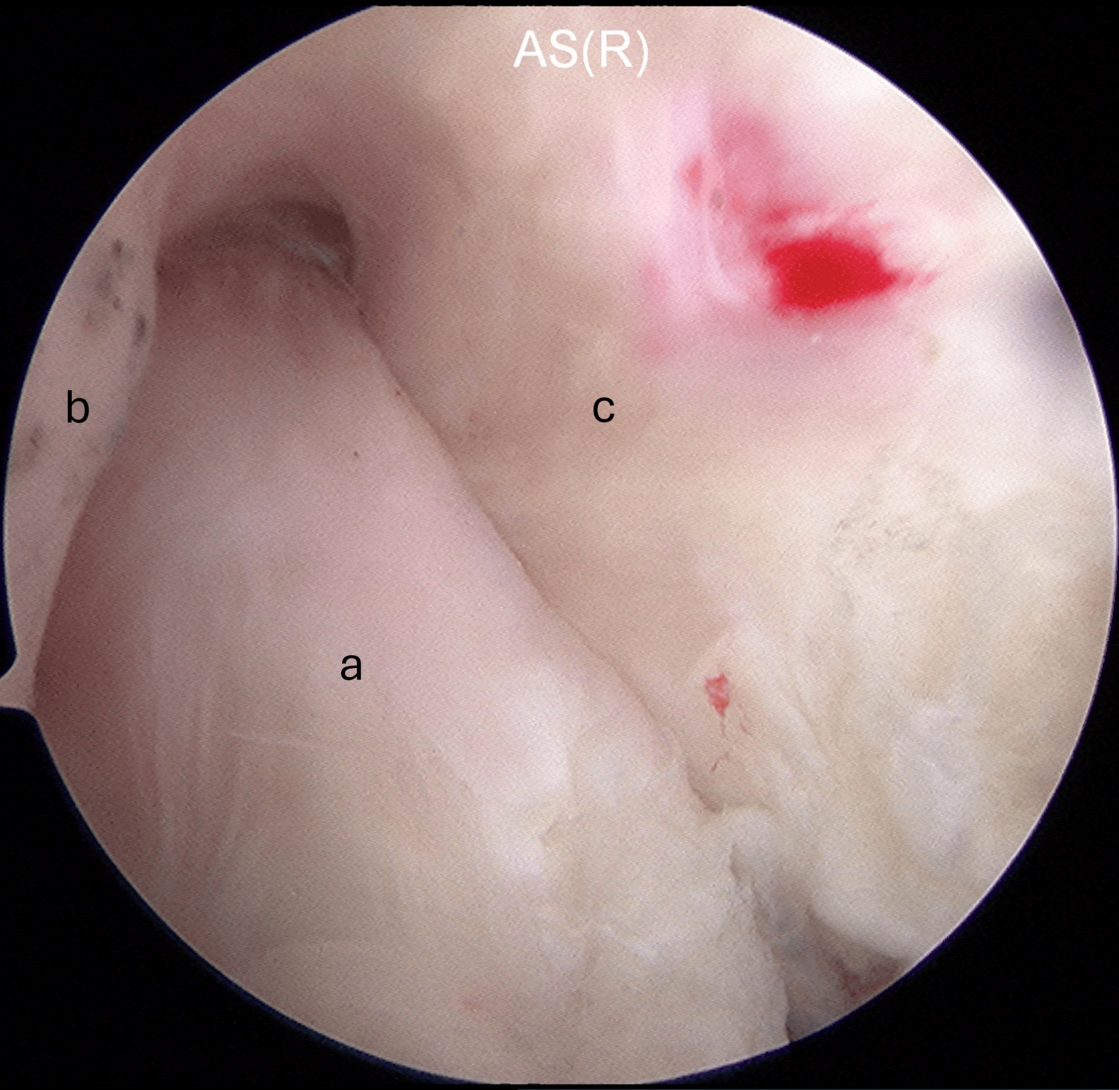
The lateral wall of the intercondylar notch was shaved, but the impingement remained: (a) the graft, (b) the lateral wall of the intercondylar notch, and (c) the PCL. PCL: posterior cruciate ligament

Arthroscopic intercondylar notchplasty was performed for graft impingement. However, notchplasty had its limitations, and the impingement remained (Figure 4).

**Figure 5:**
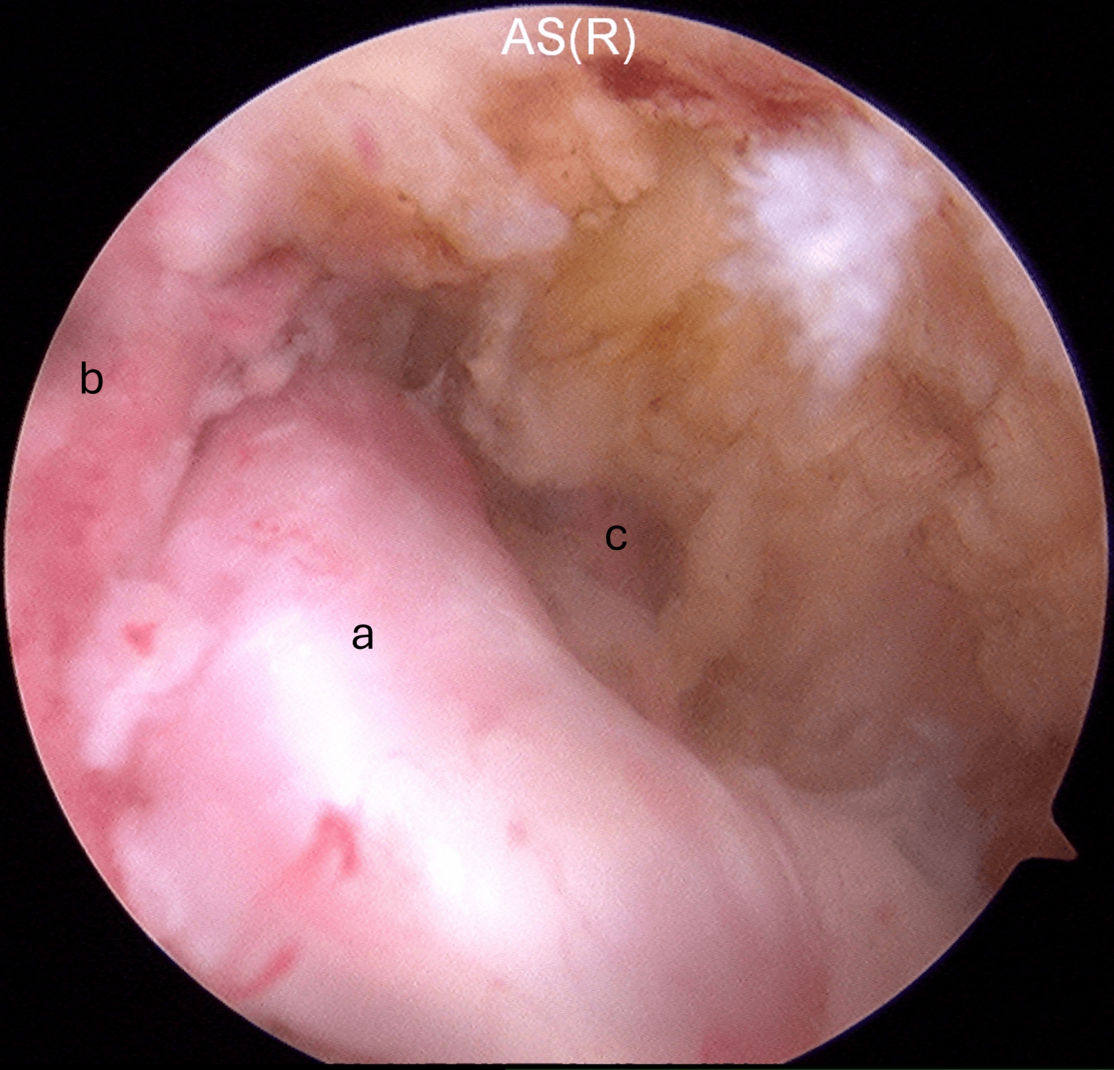
Scar tissue in front of the PCL was excised to reduce impingement: (a) the graft, (b) the lateral wall of the intercondylar notch, and (c) the PCL. PCL: posterior cruciate ligament

Moreover, the PCL was thickened with front scar tissue and was putting pressure on the ACL. Therefore, this scar tissue was resected. Consequently, the impingement nearly disappeared (Figure 5).

**Figure 6:**
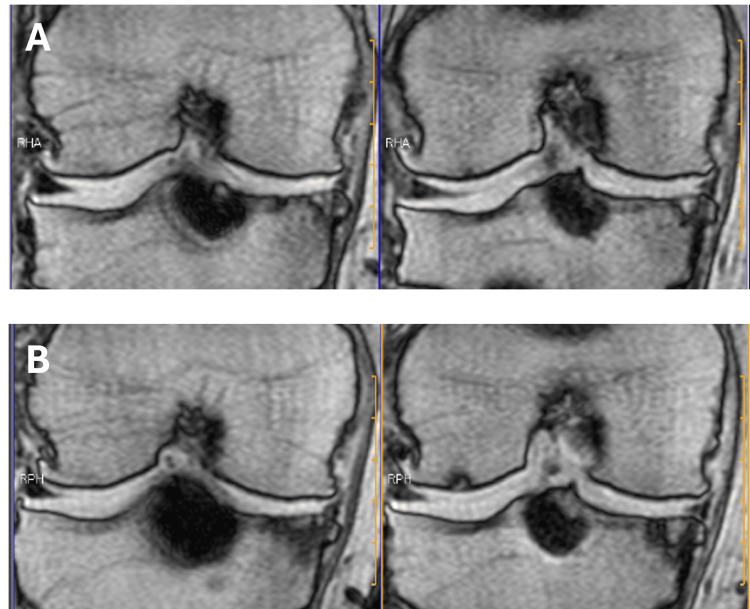
MRI showed that the intercondylar notch was well formed after arthroscopic surgery: (A) before arthroscopic surgery and (B) one year after arthroscopic surgery. MRI: magnetic resonance imaging

After surgery, pain and catching during knee extension loading that had been observed before surgery almost disappeared. After one year of surgery, the range of motion of the right knee joint was observed to be 0-140 degrees. The anterior drawer test and Lachman test were negative. The anterior movement distances measured using the KT-1000 knee joint meter were 7 and 4 mm on the right and left sides, respectively. Pain and stiffness improved after the surgery. One year after the surgery, the MRI showed good formation of the intercondylar notch (Figure 6).

**Figure 7:**
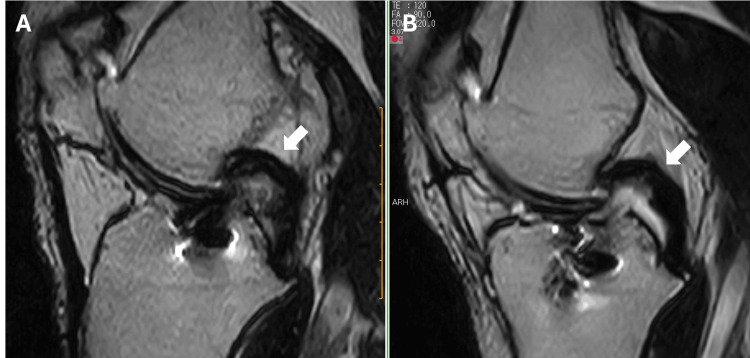
MRI showed that the front scar tissue of PCL was removed and the PCL buckling (white arrow) was reduced after arthroscopic surgery: (A) before arthroscopic surgery and (B) one year after arthroscopic surgery. MRI: magnetic resonance imaging; PCL: posterior cruciate ligament

The front scar tissue of the PCL was removed, and PCL buckling was reduced (Figure 7).

**Figure 8:**
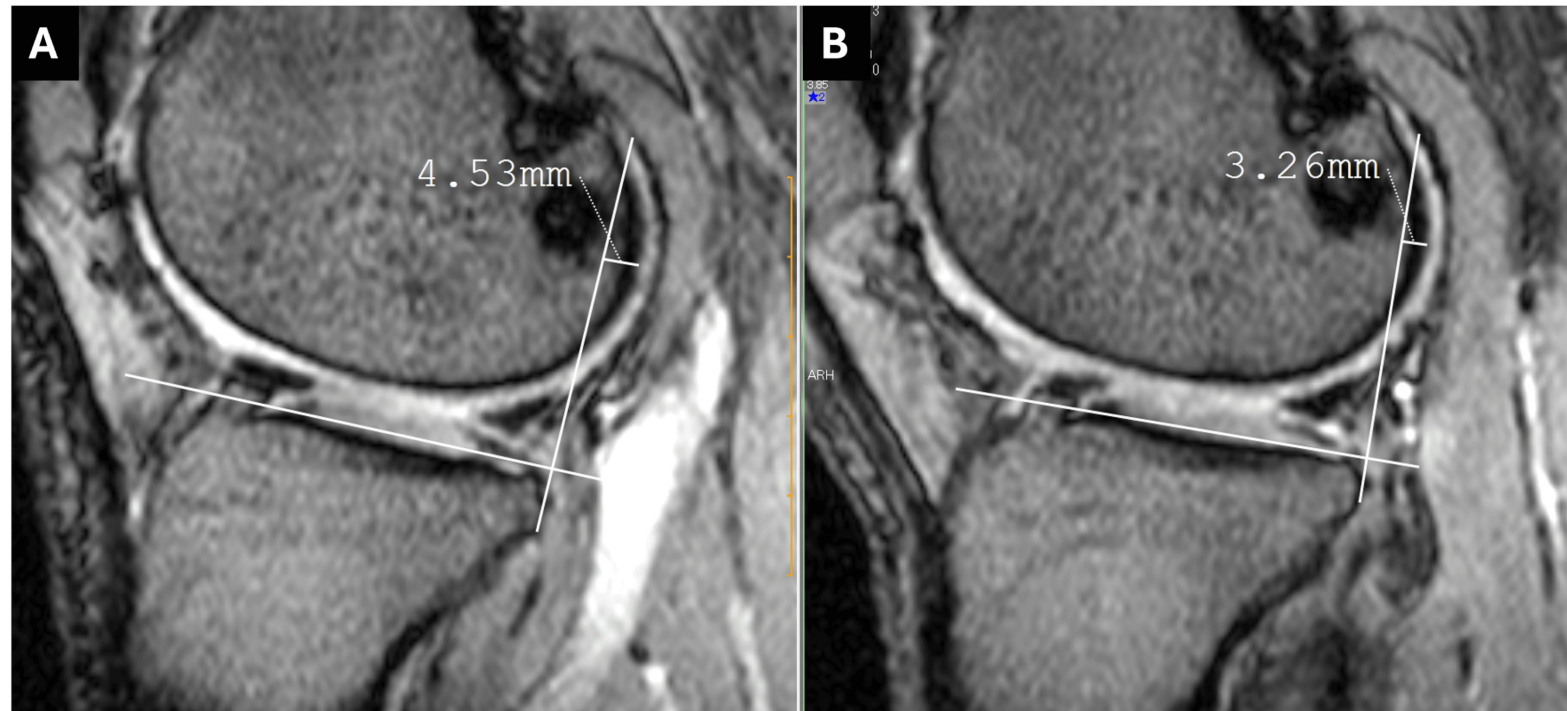
MRI showed that the distance between the rearmost part of the lateral femoral condyle and the posterior edge of the lateral tibial plateau was 1.27 mm shorter after arthroscopic surgery: (A) before arthroscopic surgery and (B) one year after arthroscopic surgery. MRI: magnetic resonance imaging

In addition, ATS was slightly reduced (Figure 8).

His symptoms have improved, and he is now able to jog.

## Discussion

In this case, PCL buckling was observed six years after ACL reconstruction. Polat et al. reported that PCL buckling can be observed even in normal knees [12]. However, in many reports, PCL buckling is considered a secondary sign of ACL tear [1-4]. Yoo and Lim reported that the degree of PCL buckling decreased after ACL reconstruction [13]. However, loose grafts do not alleviate PCL buckling [1]. In this case as well, previous ACL reconstruction probably did not resolve the PCL buckling.

ATS was also present in this case. Almekinders et al. reported ATS in some knees with ACL insufficiency and that this phenomenon may be explained by fibrosis and contracture of the posterior structures, which may inhibit posterior tibial translation and leave a permanent subluxation [6,7]. The angle of PCL buckling is an important predictor for the diagnosis of chronic ACL tears [1-4]. PCL buckling is a simple, highly reproducible, and important adjunct for the detection of ATS [14]. PCL buckling may be one of the posterior structural contractures of the knee that causes ATS.

Notchplasty is not recommended for ACL reconstruction because it alters the biomechanics of the knee after anatomical ACL reconstruction [15]. However, after ACL reconstruction, impingement of the graft with the intercondylar notch can occur, necessitating notchplasty [16]. In this case, notchplasty was performed for this impingement. However, this alone did not resolve the impingement, so we removed the anterior scar of PCL. As a result, not only PCL buckling but also ATS was reduced, and symptoms improved. Excessive preoperative ATS is associated with inferior knee stability after anatomic ACL reconstruction [17]. When excessive ATS is observed along with PCL buckling, if scar tissue anterior to the PCL is present, it may be useful to remove this scar tissue not only after ACL reconstruction but also during ACL reconstruction.

## Conclusions

To treat graft impingement after ACL reconstruction, intercondylar notchplasty and anterior PCL scar resection were performed, which not only resolved the graft impingement but also improved PCL buckling and ATS.
